# A New Generation of Dihydropyridine Calcium Channel Blockers: Photostabilization of Liquid Formulations Using Nonionic Surfactants

**DOI:** 10.3390/pharmaceutics11010028

**Published:** 2019-01-11

**Authors:** Giuseppina Ioele, Miyase Gözde Gündüz, Claudia Spatari, Michele De Luca, Fedora Grande, Gaetano Ragno

**Affiliations:** 1 Department of Pharmacy, Health and Nutritional Sciences, University of Calabria, 87036 Rende, Italy; claudia.spatari@unical.it (C.S.); michele.deluca@unical.it (M.D.L.); fedora.grande@unical.it (F.G.); gaetano.ragno@unical.it (G.R.); 2 Department of Pharmaceutical Chemistry, Faculty of Pharmacy, Hacettepe University, Ankara 06100, Turkey; miyasegunduz@yahoo.com

**Keywords:** 1,4-dihydropyridines, L- and T-type calcium channel blockers, photodegradation test, light-stable formulation, micellar solution, PET containers

## Abstract

The stability profile of a new 1,4-dihydropyridine derivative (DHP), representative of a series with a hexahydroquinoline ring, was studied to design light-stable liquid formulations. This molecule, named M3, has been shown among the analogs to have a high capacity to block both L- and T-type calcium channels. The ethanol solution of the drug was subjected to a photodegradation test, in accordance with standard rules. The concentrations of the drug and its byproducts were estimated using multivariate curve resolution, applied to the spectral data collected during the test. The improvement of both the photostability and water solubility of M3 was investigated by adding the surfactant polysorbate 20 in a 1:5 ratio to aqueous solutions of the drug. These formulations were exposed to stressing light in containers of bleu polyethylene terephthalate (PET), amber PET, and covered amber PET. The best results were obtained when using the covered amber PET container, reaching a degradation percentage of the drug less than 5% after 12 h under an irradiance power of 450 W/m^2^. The stability of the compound was compared to that of nimodipine (NIM) under the same conditions.

## 1. Introduction

**Figure 1:**
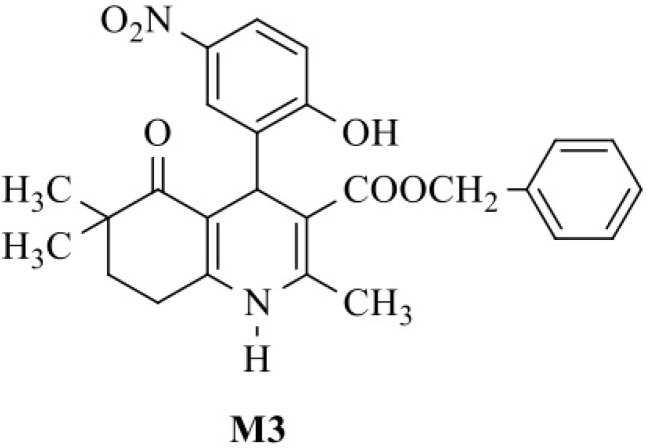
Molecular structure of M3.

Voltage-gated calcium channels play a fundamental role in essential body functions, including hormone secretion, neurotransmitter release and excitation–contraction coupling. Consequently, molecules that block these channels are of potential use in the treatment of various diseases [1]. 1,4-dihydropyridines (DHPs), the most important class of L-type calcium channel blockers, are widely used in the treatment of cardiovascular diseases, particularly hypertension and angina pectoris [2]. T-type calcium channels also attract great interest because of their potential in treating a variety of disorders, including pain and epilepsy [3,4,5,6]. Ongoing efforts to modify the DHP scaffold in order to modulate calcium channel blocking activity have led to a new class of compounds with a condensed ring system (hexahydroquinoline) [7,8,9,10]. Among these, the compound M3 (benzyl 4-(2-hydroxy-5-nitrophenyl)-2,6,6-trimethyl-5-oxo-1,4,5,6,7,8-hexahydroquinoline-3-carboxylate, see Figure 1) has been verified to block the L-type calcium channel (Ca_v_1.2), the main target of DHPs, but also the T-type calcium channel isoform Ca_v_3.1. It was concluded that this compound could serve as a new scaffold for the treatment of hypertension by reducing aldosterone secretion through the inhibition of T-type calcium channel along with the well-known antihypertensive effect by the inhibition of L-type calcium channel [7].

It is well known that DHP drugs show a high sensitivity to light, generally undergoing oxidation of the dihydropyridine ring, as shown in Figure 1 [11,12] and, in some cases, the formation of secondary photoproducts. The reaction is initiated by the transfer of the proton, probably to the solvent, from the excited single. Many DHPs present a nitro-group on the phenyl ring which accelerates the oxidation process, favoring the delocalization of the negative charge [13]. The stability seems to increase with the presence of one or more fluorine groups on the phenyl ring, while the presence of chlorine groups increases the degradation process [14]. In any case, the photodegradation process involves the total loss of the therapeutic effect. Unfortunately, it is also known that the photodegradation of the DHP drugs leads to a significant production of singlet oxygen, superoxide, or both of them, which in most cases are responsible for photosensitive/phototoxic effects [14,15,16].

Several methods have been proposed for the determination of DHPs and of the respective photoproducts [17,18,19,20,21] in bulk or pharmaceuticals. The light sensitivity is a real problem that makes it difficult to prepare liquid formulations of DHPs, which are currently dispensed in solid pharmaceutical forms.

In our previous study, the influence on the light sensitivity of different substituents on both benzene and pyridinic rings has been evaluated through a quantitative structure–property relationship (QSPR) model, which correlated the photodegradation rate against hydrophobic, electronic and steric descriptors [22]. According to these studies, we investigated the stability of M3 with the aim of realizing light-stable liquid formulations. Various approaches have been proposed to protect the DHP drugs from light or oxidation. In recent years, the incorporation of DHPs in liposomes and cyclodextrins (CDs) have been studied to increase their photostability, reporting significant results [23,24]. In fact, both these supramolecular systems have shown good ability to improve not only chemical stability but also water solubility and bioavailability for several molecules [25].

Since M3 was found to be insoluble in water, the increase of both photostability and aqueous solubility was investigated by entrapping the compound in CDs or micellar systems. The use of CDs has already been dealt with in previous studies on DHPs currently on the market [25] whereas the polyethylene glycol sorbitan monolaurate, Tween^®^ 20 (T20), was tested as surfactant. T20 is a nonionic surfactant derived from ethoxylated sorbitan esterified with lauric acid. This compound has shown high stability and relative non-toxicity in several foods, and biotechnical and pharmaceutical applications [26,27]. Its ability to improve drug water solubility and emulsion formulations is especially applied in the pharmaceutical field [28]. Because of their structure, which limits the presence of water in the internal sites, micelles formed by nonionic amphipathic surfactants like T20 provide a favorable environment to host amphiphilic drugs [29].

The further photoprotection of these aqueous formulations was tested, using containers of different materials such as quartz, bleu polyethylene terephthalate (PET), amber PET, and covered amber PET as suggested in a previous study [30]. The light-stability of M3, in aqueous and surfactant solutions, was compared with that of nimodipine (NIM), a known DHP that is commercially available. All the prepared formulations were subjected to photodegradation tests, in accordance with the ICH international rules [31]. The kinetic degradation parameters of M3 and NIM were calculated using the multivariate curve resolution–alternating least squares (MCR-ALS) technique [32,33]. The MCR method was selected for its ability to describe the photodegradation kinetics well and determine the concentration profiles of the photoproducts without any previous separation [34,35,36,37].

## 2. Materials and Methods

### 2.1. Chemicals

The synthesis of M3 has been described previously [7]. Briefly, an equimolar amount of 4,4-dimethyl-1,3-cyclohexanedione, 5-nitrosalicylaldehyde, benzyl acetoacetate and an excess of ammonium acetate were heated in methanol under microwave irradiation. After the reaction was completed, the reaction mixture was poured into ice water, and the obtained precipitate was filtered and crystallized from methanol water.

β-cyclodextrin (βCD), methyl-β-cyclodextrin (mβCD), hydroxyl-propyl-β-cyclodextrin (hpβCD), T20 and NIM were purchased from Sigma-Aldrich (Darmstadt, Germany); ethanol and methanol from J.T. Baker (Deventer, Holland). All chemicals were used without further purification. The following containers, used for the photoprotection tests, were kindly provided by Flower Tales (Milan, Italy): blue PET (thickness of 0.75 mm), amber PET (thickness of 0.8 mm) and amber covered PET (thickness of 1.2 mm).

### 2.2. Instruments

Photodegradation tests were performed using a light cabinet Suntest CPS+ (Heraeus, Milan, Italy) according to the ICH guidelines [31]. A Xenon lamp inside the cabinet and an electronic device were provided to measure and control the irradiation power. The system was also able to check the temperature inside the cabinet. The samples were irradiated according to the ID65 standard of the ICH rules.

The photodegradation experiments were monitored by UV analysis at sequential exposure times. UV spectra were recorded by a Perkin-Elmer Lambda 40P spectrophotometer (Artisan Technology Group, Mercury Drive Champaign, IL, USA) under the following conditions: λ range 200–450 nm, scan rate 1 nm s^−1^; time response 1 s; spectral band 1 nm.

Spectral data were elaborated using a multivariate approach. UV spectra were acquired by UV WinLab^®^ (Perkin-Elmer, Boston, MA, USA) and the multivariate analysis was performed using the Matlab^®^ computer environment software (Mathwork Inc., version 7, Natick, MA, USA).

### 2.3. Standard Solutions

Since M3 was found to be practically insoluble in water, standard solutions of M3 and NIM were prepared by solubilizing the compounds in ethanol at a concentration of about 20.0 μg mL^−1^.

### 2.4. Preparation of the Drug–Cyclodextrin (CD) Complex

The drug–CD complexes were prepared by optimizing a method previously described by Ioele et al. [38], which tested βCD, mβCD and hpβCD. The best percentage inclusion of DHP into the CD cavity was verified by preparing the complexes in different concentration ratios of drug to CD, namely 1:1, 1:2 and 2:1. These experiments were carried out as described by Higuchi and Connors [39].

When the complex was prepared in 1:1 molar ratio, 2 mg of M3 were dissolved in 2 mL of a CD ethanol solution at a concentration of 5 × 10^−4^ M, corresponding to βCD 49 mg or mβCD 132 mg or hpβCD 73 mg in ethanol 100 mL. A total of 10.0 mL of Britton–Robinson buffer pH 6.57 (0.04 M phosphoric acid, 0.04 M acetic acid, 0.04 M boric acid, 0.2 M NaOH) were added to this solution under stirring (120 rpm) for 24 h at 37 °C in a closed glass flask. The solution was filtered through a 0.45 μm filter and stored for 24 h at a temperature of 4 °C and finally used for analysis. During the photodegradation experiments, the spectrophotometric analysis was performed by diluting the sample (about 20.0 μg mL^−1^) at each established interval.

### 2.5. Preparation of Micellar Solutions

The micellar solutions were prepared using T20 with a concentration ratio of DHP to surfactant of 1:5. 9 mg of M3 (2 × 10^−3^ M) and 0.12 g of T20, solubilized in ethanol 1 mL. This solution was diluted in 10 mL of water and stirred (120 rpm for 24 h at 37 °C) in a closed glass flask. After that, the micellar system was filtered through a 0.45 μm filter and used for analysis after 1 h. The absorbance spectrum was recorded at each irradiation time after diluting the sample at about 20.0 μg mL^−1^ of M3. The micellar solution of NIM was prepared by adopting the same procedure and drug concentration.

### 2.6. Photodegradation Studies

Photodegradation tests were performed in the Suntest CPS+. According to the ID65 standard of ICH rules [31], the samples were irradiated under a λ range between 300 and 800 nm, fixing the irradiance power at 450 W/m^2^, corresponding to 27 kJ m^−2^ min^−1^, at a constant temperature of 25 °C. The samples were maintained in the cabinet at a distance of 20 cm from the light source. The spectra were recorded at time 0 and at the sequential time points: 1, 3, 5, 10, 15, 20, 25, 30, 35, 40, 45, 50, 55, 60, 70, 80, 90, 100, 110, 120, 130, 140, 150, 160, 170, 180, 190, 200, 230, 260, 300, 360 and 420 min.

### 2.7. Chemometric Elaboration

UV spectra, collected during the photodegradation experiments, were processed by MCR-ALS. The application of this algorithm decomposes the matrices of the experimental data into the contributions of the single components, thus estimating the number of components and the relative spectra as well as the concentration profiles and the rate constants (k) of the kinetic process.

## 3. Results

### 3.1. Photodegradation in Ethanol Solution

**Figure 2:**
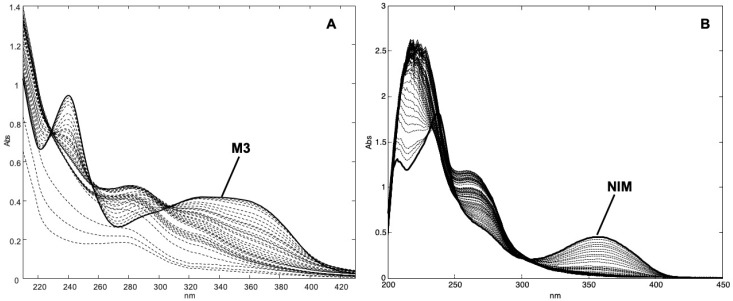
UV spectra recorded during the photodegradation experiments on ethanol solutions of M3 (**A**) and nimodipine (NIM) (**B**).

Due to the low solubility in water, the photodegradation of the tested compounds was first executed in ethanol solutions, at a concentration of 20.03 and 20.11 μg mL^−1^ for M3 and NIM, respectively, as above described. These solutions were placed in quartz cuvettes and subjected to stressing light. The absorbance spectra from 200 to 450 nm were recorded before the experiment (*t* = 0 min) and at time intervals up to 7 h of exposure. Figure 2 shows the sequences of the recorded absorbance spectra of M3 (Figure 2A) and NIM (Figure 2B).

The data were elaborated by MCR in such a way as to predict the number of photoproducts as well as their spectra and concentration profiles. The degradation process followed a pseudo second-order kinetics, described by the equation:

1/[% DHP] = *k* · *t* + 0.01

where % DHP is the percentage value of the residual drug concentration, *k* is the photodegradation rate constant, *t* is the time (expressed in minutes) and 0.01 is the reciprocal value of the starting concentration percentage (100%).

The parameter *t*_0.1_ (time to obtain a 10% degradation) was chosen as a criterion to compare the behavior of the formulations during the photodegradation experiments. The adoption of this parameter is justified by considering a pharmaceutical formulation useless when the bioavailable quantity of the active ingredient falls below 90%. The parameter t_0.5_ was also measured to compare the shelf life of the tested formulations. The calculated kinetic parameters are listed in Table 1 which summarizes the values collected in all the photodegradation experiments. The presented data were the average of six experiments. Relative standard deviation values of all points were within the range 1.94–5.76%.

**Figure 3:**
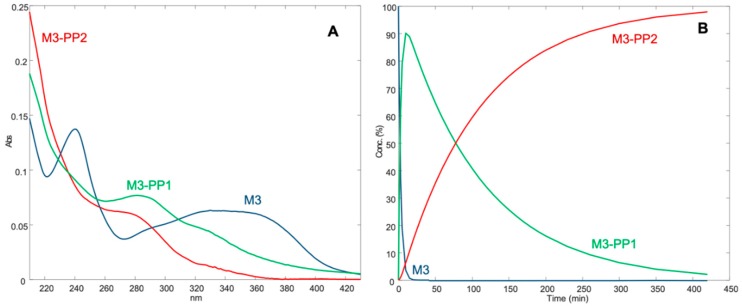

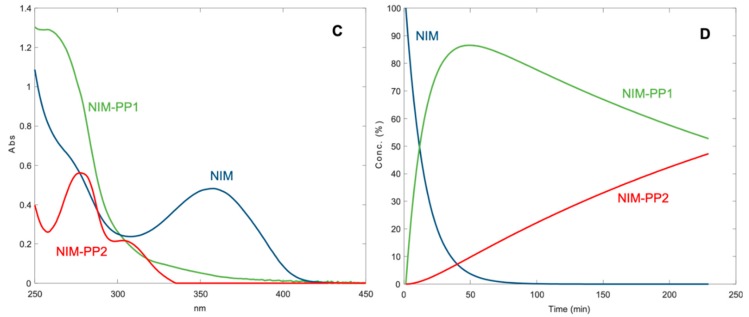
Spectra (**A**) and concentration profiles (**B**) for M3 and its photoproducts; spectra (**C**) and concentration profiles (**D**) for NIM and its photoproducts.

The results from MCR analysis are shown in Figure 3. The elaboration of the data assumed the formation of two photoproducts for both compounds. The predicted absorbance spectra and the concentration profiles of the photoproducts of M3, namely M3-PP1 and M3-PP2, are shown in part A and B of Figure 3, respectively. In the same Figure, the spectra (C) and concentration profiles (D) for NIM and its photoproducts, NIM-PP1 and NIM-PP2, confirmed the results reported in the literature [19,25].

### 3.2. Photodegradation in Cyclodextrin Complex

In order to provide an aqueous formulation of M3, the preparation of a water-soluble complex with cyclodextrin was initially undertaken. According to our previous study [38], several DHP-CD complexes were prepared and different concentration ratios M3:CD were tested. The higher solubility values were obtained when the M3-CD complexes were prepared with a 1:1 ratio using hpβCD and 2:1 using βCD and mβCD. The entrapment efficiency was calculated by spectrophotometry and the values, expressed as a percentage, are reported in Table 2.

**Figure 4:**
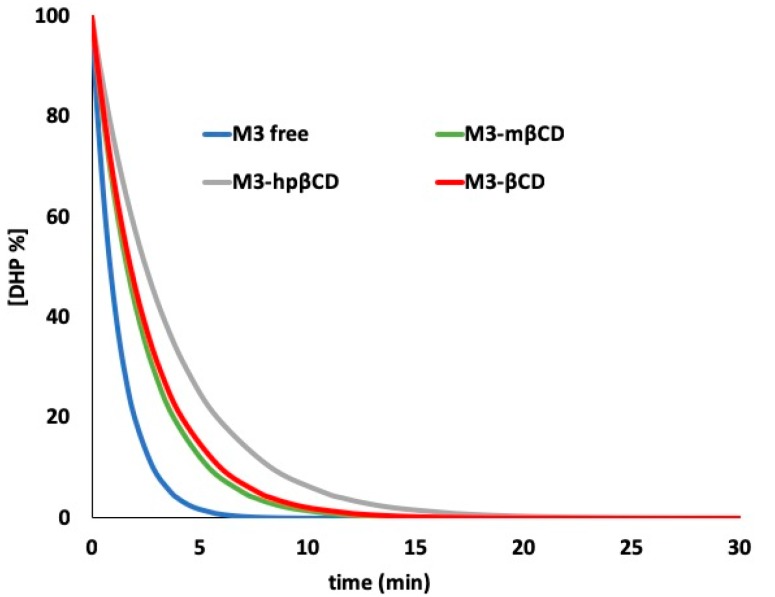
Photodegradation of M3 in the cyclodextrin complexes.

These three complexes were exposed to light. Figure 4 shows the photodegradation profiles of M3 complexed with the different CDs, compared with the degradation of the ethanol solution of the free drug. This graph was obtained by tracing the percentage concentration of the residual drug with respect to the irradiation time expressed as minutes.

The MCR analysis applied to the spectral data showed a pseudo second-order degradation kinetics for the degradation of all the M3-CD complexes. Table 2 lists the kinetic degradation parameters, showing entrapment efficiency values below 30% in all complexes. This result confirmed the difficulty in the drug entrapment phase, already described for NIM in our previous work [25].

### 3.3. Photodegradation in Micellar Solution

**Figure 5:**
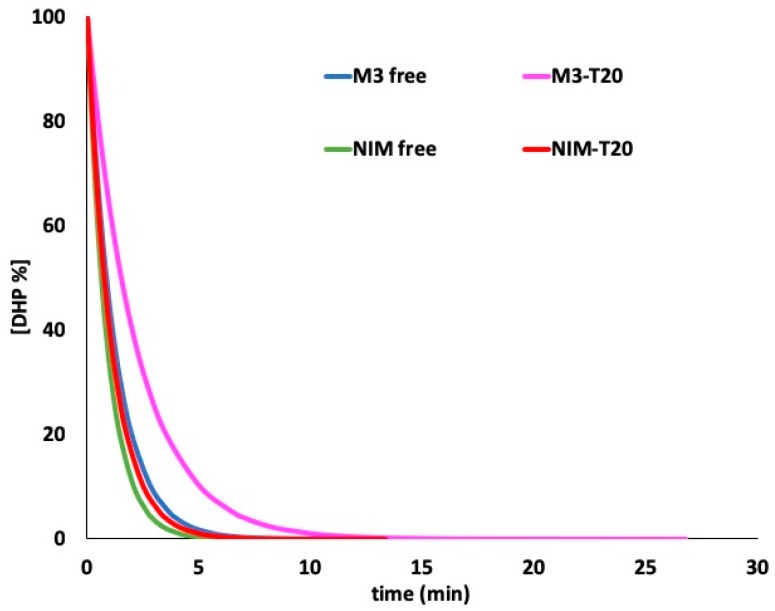
Photodegradation of M3 and NIM in ethanol and surfactant solutions.

Micellar solutions of M3 and NIM were prepared as described above, obtaining a clear improvement in the solubility for both drugs. The concentration in the micellar solutions was calculated to be 92% of the starting concentration for M3 and 89% for NIM, respectively. These solutions were exposed to light in quartz cuvettes. Figure 5 shows the photodegradation profiles of M3 and NIM in a T20 solution and compared with those of the free drugs. MCR analysis was applied to the spectra to calculate the kinetic parameters, reported in Table 1. The degradation profiles followed a pseudo second-order kinetics.

### 3.4. Photodegradation Studies of DHPs in PET Containers

**Figure 6:**
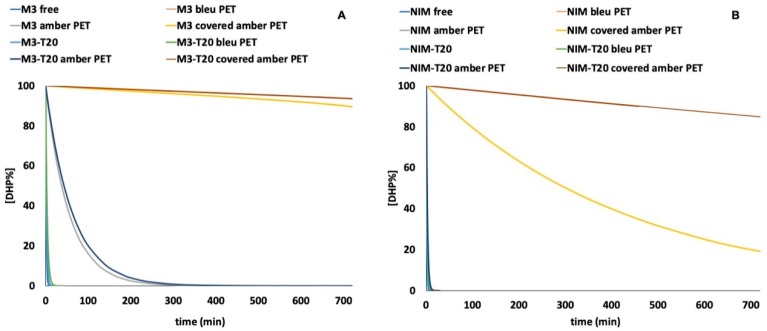
Photodegradation of M3-T20 (**A**) and NIM-T20 (**B**) in different PET containers.

The photostability of the drug–T20 formulations was then tested by placing them in different PET containers such as amber PET, covered amber PET and blue PET. In these experiments, the light exposure was prolonged up to 720 min. Figure 6 shows the photodegradation profiles of M3 (A) and NIM (B) in the tested containers, compared with the results obtained from the ethanol solutions of the free drugs. The kinetic parameters calculated by MCR are listed in Table 1.

The analysis of the photodegradation profile showed a pseudo second-order degradation kinetics. The zero-order kinetics was calculated when the M3 and NIM ethanol solutions, M3-T20 and NIM-T20 were exposed to light in the covered amber PET containers, using the following equation:

[% DHP] = −*k* · *t* + 100

where % DHP is the percentage value of the residual drug concentration, *k* is the photodegradation rate constant, *t* is the time (expressed in minutes) and 100 is the percentage of the starting concentration.

## 4. Discussion

A photodegradation study was performed on a newly synthesized DHP, named M3, and compared with those obtained from the congener NIM. This drug showed promising effects on L- and T-type calcium channels, evaluated in the Department of Physiology and Pharmacology, Hotchkiss Brain Institute, University of Calgary, Canada. This work was part of a larger project involving the entire class of DHPs, aimed at designing light-stable liquid formulations. Precisely because of their poor water solubility and light instability, these drugs are dispensed in solid formulations.

The MCR analysis (Figure 3) applied to the spectra from the photodegradation experiments showed the very high instability of the compounds in ethanol solutions, with a t_0.1_ value of 2.84 min and 2.07 min for M3 and NIM, respectively. When M3 was exposed to light, the complete disappearance of the characteristic peak in the region 320–360 nm was found after just 10 min. A first photoproduct (M3-PP1) appeared after a few seconds of light exposure, which in turn transformed to M3-PP2 in the next 30 min. According to the literature [13], it could be hypothesized that M3-PP1 corresponds to the pyridine derivative and the formation of M3-PP2 is due to the reduction of the NO_2_ group on the phenyl ring.

The improvement of both the photostability and aqueous solubility of M3 was investigated by entrapping the compound in cyclodextrins. The incorporation percentage was measured by spectrophotometry and the percentage efficiency values are reported in Table 2. The solubility of the drug was verified to increase in all the prepared complexes, but the obtained values were not considered satisfactory. The poor results were attributed to the low drug encapsulation owing to the relevant molecular volume of the drug. The best performance was reached when the drug was incorporated in hpβCD, obtaining an encapsulation percentage of 29.7%. Unfortunately, when this complex was exposed to light, an unsatisfactory t_0.1_ value of 8.33 min was measured. For this reason, this system could be used to increase the DHP’s solubility in water but its use in the development of light-stable liquid formulations is not recommended. Moreover, as described in a previous study [25], when NIM was incorporated with mβCD at a ratio of 1:1, the values of k and t_0.1_ were calculated to be 0.0102 and 4.5 min (*R*^2^ = 0.9976), respectively. The encapsulation difficulties were attributed to the large chemical groups on both molecular rings of this drug.

In the last years, the preparation of micellar solutions of drugs have become popular as drug delivery systems [26,27]. The use of surfactants or amphiphilic substances helps the formation of micelles in aqueous solution, with the hydrophilic “head” regions in contact with the surrounding solvent and the core able to include hydrophobic compounds. This approach was pursued to entrap the hydrophobic DHPs and thus increase their water solubility. Thanks to its common use in food preparation, T20 was selected as the surfactant for the preparation of the micellar solutions. The concentration of the drugs entrapped in the micelles, spectrophotometrically measured, was satisfactory, resulting in a high increase of solubility for both the drugs.

The prepared micellar solutions were then subjected to the photodegradation test. When the MCR technique was applied to the spectra from the degradation experiments, the same photoproducts detected during the light exposure of the ethanol solutions were detected. The kinetic parameters showed a clear decrease in the photodegradation rate, demonstrating a higher stability of the compounds entrapped in the micelles with respect to the free drugs. The *t*_0.1_ value calculated for M3-T20 and NIM-T20 were 5.05 and 2.52 min, respectively, as reported in Figure 5 and listed in Table 1. The persistent light sensitivity of the DHPs was probably due to oxidation reactions, favored by the water environment.

The improvement in water solubility stimulated the subsequent design of a packaging system able to ensure the photoprotection of the micellar solutions. In a previous work [30], significant photoprotection for the same DHPs was obtained with the use of PET containers. The best result was achieved when a hydroalcoholic solution of felodipine was stored in a transparent blue PET container, showing almost complete stabilization for up to 6 h under stressing light exposure. In our study, the micellar solutions of M3 and NIM were placed in different PET containers and exposed to light. The test was also performed on the ethanol solutions of the free drugs. As depicted in Figure 6, all the containers tested showed a significant increase in the light stability for both the compounds. The best photoprotection for M3 was obtained by using the amber and covered amber PET containers, with a percentage degradation of the drug lower than 5% after 12 h under stressing irradiation in covered amber PET. Moreover, MCR elaboration showed the formation of only one photoproduct corresponding to the pyridine byproduct. Even better results were obtained with the use of T20 for the protection of NIM, which in previous experiments showed higher instability with respect to the M3 formulations. The complete light-stabilization of NIM was in fact achieved in the covered amber PET containers.

## 5. Conclusions

In this work, some approaches have been proposed to develop light-stable liquid formulations for a compound representing a new generation of DHPs with non-selective activity on L- and T-type calcium channels. The goal of the work was the improvement of both solubility in the water and light-stability of these compounds. No satisfactory results were reached when the drug was entrapped in cyclodextrin matrices, due to the very low drug encapsulation (<30%). On the contrary, the use of surfactants considerably increased the DHP solubility in water. Satisfactory light-stability of this formulation was furthermore obtained by adopting different PET containers, able to absorb the wavelengths below 300 nm, which promoted the photodegradation of these molecules. The proposed systems demonstrated a remarkable capacity for photoprotection and their use could be extended to other DHPs in order to develop liquid formulations of these photosensitive drugs.

## Figures and Tables

**Table 1:** Degradation kinetic parameters calculated for M3 and NIM formulations.

Sample	Container	*k* (×10^−3^)	*t*_0.1_ (min)	*t*_0.5_ (min)	*R* ^2^
M3 free	quartz	0.351	2.84	28.49	0.992
	blue PET	0.132	7.58	75.76	0.999
	amber PET	0.008	125.00	-	0.999
	covered amber PET	-	-	-	-
M3-T20	quartz	0.198	5.05	50.51	0.969
	blue PET	0.125	8.00	80.00	0.999
	amber PET	0.007	142.86	-	0.996
	covered amber PET	-	-	-	-
NIM free	quartz	0.481	2.07	20.79	0.999
	blue PET	0.291	3.44	34.36	0.996
	amber PET	0.184	5.44	54.35	0.999
	covered amber PET	0.066	-	-	0.989
NIM-T20	quartz	0.397	2.52	25.19	0.992
	blue PET	0.241	4.15	41.49	0.991
	amber PET	0.181	5.52	55.25	0.991
	covered amber PET	0.006	-	-	-

**Table 2:** Kinetic parameters of M3-CD complexes.

Samples	% Entrapment Efficiency	*k* (×10^−3^)	*t*_0.1_ (min)	*R* ^2^
M3 free	-	0.351	2.84	0.992
M3-mβCD	15.9	0.184	5.43	0.992
M3-hpβCD	29.7	0.120	8.33	0.988
M3-βCD	18.3	0.167	5.98	0.985
